# Costimulation to enhance the antitumor activity of CD19 eng T cells

**DOI:** 10.1186/2051-1426-3-S2-P58

**Published:** 2015-11-04

**Authors:** Mireya Velasquez, David Torres, Aarohi Thakkar, Sunitha Kakarla, Carolyn Arber Barth, Xiao-Tong Song, Stephen Gottschalk

**Affiliations:** 1 Baylor College of Medicine, Center for Cell and Gene Therapy, Houston, TX, USA; 2 Baylor College of Medicine, Houston, TX, USA

## Background

Immunotherapy with T cells or bispecific T cell engagers is one promising approach to improve outcomes for patients with CD19-positive hematological malignancies. We had previously shown that T cells expressing bispecific T cell engagers that recognize CD19 and CD3 (CD19-ENG T cells) are activated in an antigen dependent manner, recruit resident T cells to tumors, and have anti-tumor activity in preclinical models. In this project we now wanted to evaluate if provision of co-stimulation enhances the effector function of CD19-ENG T cells by expressing CD80 and 41BBL on their cell surface (CD19 ENG/costim T cells).

## Methods

CD19-ENG T cells were generated by transducing T cells with a retroviral vector encoding a CD19-specific T cell engager, and CD19-ENG/Costim T cells were generated by double transducing T cells with the previous construct and a 2^nd^ retroviral vector encoding the costimulatory molecules 41BBL and CD80. The effector function of the generated T cells was evaluated *in vitro* and in a xenograft model.

## Results

CD19-ENG and CD19-ENG/Costim T cells recognized CD19+ lymphoma (Daudi, Raji) and acute leukemia (BV173) cells as judged by IFN-g secretion. Both ENG T cell populations produced IL-2 in the presence of CD19-positive targets expressing CD80 and CD86 (Daudi and Raji). However, CD19-ENG/Costim T cells produced higher levels of IL-2 in comparison to CD19-ENG T cells after stimulation with BV173 (CD19+CD80-CD86-). ENG and ENG/Costim T cells specific for an irrelevant antigen (EphA2) did not produce cytokines, confirming antigen dependence. Specificity was confirmed in cytotoxicity assays. *In vivo* anti-tumor activity of CD19-ENGand CD19-ENG/Costim T cells was assessed in a BV173/NSG xenograft model. First we determined the minimal required CD19-ENG T cell dose to observe anti-tumor effects. Three injections of 1x10^7^ CD19-ENG T cells cured 5/5 mice, one injection 2/5 mice, and one dose of 1x10^6^ CD19-ENG T cells had no anti-tumor effects. In contrast one dose of 1x10^6^ or 1x10^7^ CD19-ENG/Costim T cells cured 5/5 mice. ENG T cells or ENG/Costim T cells recognizing EphA2 had no anti-tumor effects.

## Conclusions

Provision of co-stimulation increases the effector function of CD19-ENG T cells resulting in enhanced anti-tumor activity *in vivo*. Genetically modifying T cells to express engagers and additional molecules to enhance their effector function may present a promising alternative to current CD19-targeted immunotherapies.

